# Greater periventricular white matter hyperintensity severity in basilar artery branch atheromatous disease

**DOI:** 10.1186/s12883-017-0918-y

**Published:** 2017-07-17

**Authors:** Po-Chen Lin, Feng-Chi Chang, Hui-Chi Huang, Jui-Yao Tsai, Yung-Yang Lin, Chih-Ping Chung

**Affiliations:** 1 Department of Neurology, Taipei Veterans General Hospital, No. 201 Sec.2, Shihpai Road, Peitou, Taipei, 11217 Taiwan; 2 National Yang Ming University, Taipei, Taiwan; 3 Department of Radiology, Taipei Veterans General Hospital, Taipei, Taiwan; 4 National Taipei University of Nursing and Health Sciences, Taipei, Taiwan

**Keywords:** White matter hyperintensity, Branch atheromatous disease, Posterior circulation ischemic stroke, Basilar artery

## Abstract

**Background:**

Basilar artery branch atheromatous disease (BABAD), in which basilar artery atheroma occludes penetrating arteries at their origin, is a common etiology of posterior circulation stroke (PCS). It is currently unknown whether white matter hyperintensity(WMH), a marker of small vessel disease(SVD), is associated with BABAD.

**Methods:**

The present study analyzed data from patients with PCS who were enrolled in the Taipei Veterans General Hospital Stroke Registry between January 1, 2010 and February 28, 2014. WMH severity was rated using the Scheltens scale. We used multivariate analyses to: (1) compare the severity of WMH between patients with BABAD, patients with large-artery > 50% atherosclerotic stenosis-related PCS(LAA), and non-stroke subjects(NS); and (2) evaluate the relationship between WMH severity and the 3-month prognosis of patients with BABAD.

**Results:**

The study pool included 151 BABAD, 97 LAA, and 78 non-stroke patients. Multivariate analyses adjusting for age, sex, and vascular risk factors showed that compared to patients with LAA [Odds ratio(OR) = 0.51, *p* = 0.037] and NS (OR = 0.40, *p =* 0.004), patients with BABAD (OR = 1) had greater WMH severity (score ≥ 50th percentile) in periventricular, but not subcortical, regions. Moreover, greater periventricular WMH severity predicted poor 3-month functional outcomes (modified Rankin Scale > 3) with an OR of 3.21 (*p* = 0.028) in BABAD patients.

**Conclusions:**

We are the first to show a significant association between WMH and BABAD that is independent of vascular risk factors and atherosclerotic large-artery disease. Our results suggest that small vessel abnormalities other than lipohyalinosis may be involved in BABAD pathophysiology. A future management strategy should include both large and small vessel protection.

## Background

The two types of posterior circulation stroke (PCS) that are associated with atherosclerotic large-artery disease are (1) ischemic stroke with associated large-artery (vertebrobasilar artery, VBA) > 50% atherosclerotic stenosis (LAA) and (2) branch atheromatous disease (BAD) [1, 2]. In BAD, small vessels (i.e., penetrating arteries) are occluded at their origin by microatheroma or plaque within large parent arteries (e.g., basilar artery). We recently showed that basilar artery BAD (BABAD) was the most common etiology of PCS in our stroke registry [2]. BABAD typically causes small infarcts within the paramedian pons without significant VBA stenosis. Consequently, BABAD may be misdiagnosed as lacunar infarction, which is caused by the occlusion of small vessels resulting from lipohyalinosis [3, 4]. The seminal pathological studies of Fisher and Caplan [3] first described BABAD and distinguished it from lacunar infarction caused by small vessel lipohyalinotic degeneration. Decades later, high-resolution MRI is available to examine microatheromas or shallow plaques within the BA that occlude penetrating arteries at their origin and cause small infarcts within the paramedian brainstem [5, 6]. However, it is currently unknown whether small vessel abnormalities other than lipohyalinosis are involved in BAD mechanisms.

White matter hyperintensity (WMH) is commonly found in brain MR images of the elderly [7, 8]. Several small vessel abnormalities, including impaired cerebrovascular vasoreactivity, endothelial dysfunction, decreased blood flow to cerebral white matter, collagenosis of cerebral venules, and blood-brain-barrier (BBB) impairment, have been observed in subjects with WMH [7–13]. WMH has been classified as a marker of small vessel disease (SVD). The association between WMH and BAD has not been previously studied. Thus, the present study aimed to (1) compare the severity of WMH between patients with BABAD, patients with LAA, and non-stroke subjects (NS) and (2) examine the relationship between WMH severity and the 3-month prognosis of patients with BABAD. We hypothesized that in addition to large-artery atherosclerosis, BAD pathophysiology may also involve small vessel abnormalities, which would be reflected by (1) greater WMH severity in the BABAD group compared to the NS and LAA groups or (2) an association between WMH severity and the 3-month functional outcome of the BABAD group.

## Methods

### Stroke registry and stroke etiology

Since February 2009, the Taipei Veterans General Hospital Stroke Registry (TVGHSR) has collected data for all patients with acute stroke admitted to the emergency room or Neurology Department wards. Two specialized nurses, who were supervised by physicians specializing in stroke medicine, managed the patient data registry and followed up on patient outcomes. Registry data included: (1) demographic information and risk factors; (2) stroke severity at admission; (3) neurological, vascular, or cardiological assessment results acquired during hospitalization; and (4) outcomes at discharge and 3-month follow-up (via telephone). The TVGHSR data were analyzed for the present study.

Patient data were reviewed and a consensus concerning their stroke etiologies was reached by two neurologists (Drs. Chung and Lin) and one radiologist (Dr. Chang) who specialize in stroke medicine. The stroke etiology of patients with adequate investigations including brain MRI and MRA, duplex ultrasound of neck arteries, color-coded transcranial Doppler imaging, 24-h Holter monitor recording, and echocardiography, was determined. The following standardized criteria were used to classify stroke etiology LAA and BABAD [1, 2]:

1. BABAD. Vascular and cardiological studies showed the absence of large artery occlusion or severe stenosis (> 50%) in the BA or vertebral arteries (VAs) as well as cardioembolic risks. BA showed atheroma or plaque by MRI T1 (isointense) or/and T2/FLAIR (hypointense) sequence at the level of cerebral infarct. Clinical and brain imaging study were compatible with paramedian pontine/midbrain/upper medulla infarct (usually were basal-brainstem involved and wedge-shaped) due to several penetrating vessels occlusion.
2. LAA. Vascular studies showed occlusion or severe stenosis (>50%) of the relevant large artery. There should be multiple or diffusely atherosclerotic changes in the other large arteries. Abrupt cut-off of vessels was considered more likely due to embolism and was not considered for this category. Cerebral infarcts were in the territory of the large artery stenosis/occlusion larger than that of a single branch artery territory, or in distal fields.

### Study population

We retrieved data from patients who were registered in the TVGHSR between January 1, 2010 and February 28, 2014. The following inclusion criteria were applied to the selected subjects: (1) presence of acute cerebral infarcts in the territory of posterior circulation with BABAD or LAA etiology; and (2) an adequate amount of supporting assessments during admission, including brain MRI. Patients with (1) malignancy, (2) autoimmune diseases, (3) hematological diseases, or (4) simultaneous acute cerebral infarcts in the territory of anterior circulation were excluded. We also excluded patients with posterior cerebral artery territory (i.e., supratentorial) infarct to avoid difficulties with WMH grading.

NS from our outpatient clinics who received brain MRI during physical examinations were invited to join the present study. Subjects with (1) dementia, (2) severe heart disease, and (3) malignancy were not enrolled. NS demographic and vascular risk factor information was obtained from patient history records or laboratory data. All participants provided informed consent. The Institutional Review Board of Taipei Veterans General Hospital approved the present study.

### WMH assessment

All study participants (i.e., NS and patients with BABAD or LAA) were imaged with a 1.5 T MRI scanner (Excite II; GE Medical Systems, Milwaukee, WI, USA). Whole brain axial T1- and T2-weighted sequences and fluid-attenuated inversion recovery (FLAIR) pulse sequences were performed. WMHs were defined as lesions that appeared hyperintense on T2-weighted and FLAIR images and mildly hypointense or normal on T1-weighted images. Some patients with BABAD underwent both standard and high-resolution MRI examination to evaluate BA plaque. Only standard MRI datasets were used to determine the WMH rating.

To assess WMH severity and anatomical distribution, we used the semiquantitative Scheltens scale to rate WMH in periventricular and subcortical regions [14]. For this scale, the severity of hyperintensities in different brain areas is scored separately. These brain areas include three periventricular regions (i.e., frontal caps, bands, and occipital caps) and four areas of deep subcortical white matter (i.e., frontal, parietal, temporal, and occipital). The periventricular WMH rating was determined with scores of 0 = absent, 1 = ≤ 5 mm, and 2 = > 5 mm in each of the three periventricular regions, resulting in a maximum rating of 6 points. The subcortical WMH rating was determined using a 0–6-point scale related to lesion size and number. If the largest lesion in a region was <4 mm, then a score of 1 was given if there were ≤ five lesions, whereas a score of 2 was given if there were > five lesions. If the largest lesion was 4–10 mm, a score of 3 was given if there were ≤ five lesions, whereas a score of 4 was given if there were > five lesions. A score of 5 was given for very large lesions (≥ 10 mm) and a score of 6 was given for confluent lesions. Thus, in subcortical regions, the scale could produce a maximum score of 24 points. Analysis of MRI datasets was performed to determine WMH ratings by one neurologists (Dr Lin) who was well-trained in neuroimaging interpretation and blinded to the clinical characteristics of the subjects while MRI WMH rating. Intra-rater reliability was tested using a subset of 20 consecutive study subjects. The Kappa (κ) coefficient for periventricular and subcortical WMH ratings were 0.93 and 0.90, respectively.

### Statistical analyses

Analyses were performed with SPSS software (version 22.0; IBM, USA). All data are presented as mean [standard deviation (SD)] for continuous variables and number (percentage) for discrete variables. Group comparisons were made using a nonparametric Kruskal-Wallis test. Bonferroni correction was applied for pairwise group comparisons. When appropriate, a chi-square (χ2) test or Fisher’s exact test was performed for categorical variables. Multivariate analyses were used to adjust for confounding factors, such as age, sex, and vascular risk factors. Statistical significance was reached when *P* < 0.05. To adjust for confounding factors (age, sex and vascular risk factors including hypertension, diabetes mellitus, hyperlipidemia and cigarette smoking), we performed multivariate analyses. We used the 50th percentile of the WMH score as the cutoff point. To respectively compare the severity of WMH between BABAD group and NS and LAA group, we analyzed the odds ratio (OR) of greater WMH (>50th percentile of the WMH score) in NS and LAA group versus BABAD respectively.

## Results

For this study, we analyzed data from 151 patients with BABAD, 97 patients with LAA, and 78 NS. Table 1 shows infarct locations and vascular involvement in the BABAD and LAA groups. The pons was the most common location involved in BABAD and LAA (93.4% versus 50.5%). The only other BABAD infarct locations were the midbrain (4.0%) and medulla (2.6%). All BABAD-associated PCS subjects exhibited a single lesion. In the BABAD group, severe VBA stenosis (> 50%) was not present; however, severe internal carotid artery (ICA) and middle cerebral artery (MCA) stenosis were present in 43.0% and 24.5% of the subjects, respectively.

**Table 1:** The location of brain infarcts and presence of >50% atherosclerotic stenosis in the large arteries of patients with BABAD and LAA

	BABAD (*n* = 151)	LAA (*n* = 97)
Infarct locations		
Midbrain	6 (4.0%)	5 (5.2%)
Pons	141 (93.4%)	49 (50.5%)
Medulla	4 (2.6%)	12 (12.4%)
Cerebellum	0	23 (23.7%)
> 50% atherosclerotic stenosis		
VA	0	70 (89.7%)
BA	0	57 (73.1%)
ICA	65 (43.0%)	51 (65.4%)
MCA	37 (24.5%)	38 (48.7%)
Admission NIHSS	4.7 (3.9)	5.9 (6.8)

Data are presented as mean (SD) or number of patients (percentage)

*BABAD* basilar artery branch atheromatous disease; *LAA* large-artery >50% atherosclerotic stenosis; *VA* vertebral artery; *BA* basilar artery; *ICA* internal carotid artery; *MCA* middle cerebral artery; *NIHSS* NIH Stroke Scale

Table 2 presents demographic and WMH comparisons between the BABAD, LAA, and NS groups. The frequency of vascular risk factors was significantly different between groups. The BABAD and LAA groups had greater frequencies of hypertension, diabetes mellitus, and hyperlipidemia compared to the NS group, whereas the NS group had a greater frequency of cigarette smoking.

**Table 2:** Demographic and white matter hyperintensity comparisons between NS and patients with BABAD and LAA

	BABAD (*n* = 151)	LAA (*n* = 97)	NS (*n* = 78)	*p*
Age (years)	72.3 (10.6)	73.2 (11.5)	72.6 (12.0)	0.786
Sex (men)	101 (66.9%)	54 (69.2%)	69 (71.1%)	0.790
Vascular risk factors				
Hypertension	121 (80.1%)	68 (87.2%)	51 (52.6%)	<0.001
Diabetes mellitus	72 (47.7%)	38 (48.7%)	18 (18.6%)	<0.001
Hyperlipidemia	32 (21.2%)	24 (30.8%)	14 (14.4%)	0.034
Cigarette Smoking	24 (15.9%)	20 (25.7%)	30 (30.9%)	0.008
Periventricular WMH				
Total score	3.3 (1.7)	2.9 (1.9)	2.4 (2.4)	0.001^a^
Score ≥ 3 (50th percentile)	99 (65.6%)	43 (44.3%)	43 (55.1%)	0.003
Subcortical WMH				
Total score	3.2 (3.1)	2.5 (2.8)	2.6 (3.4)	0.044
Score ≥ 2 (50th percentile)	84 (55.6%)	36 (37.1%)	47 (60.3%)	0.292

*BABAD* basilar artery branch atheromatous disease; *LAA* large-artery >50% atherosclerotic stenosis; *NS* non-stroke subjects; *WMH* white matter hyperintensity

^a^ *Post-hoc* Bonferroni analysis revealed that the BABAD group had a higher periventricular WMH score than the NS group

The mean WMH score for the whole study population was 3.0 [2.0 (SD); 0–6 (range); 1 (1st quartile); 3 (2nd quartile); 5 (3rd quartile)] for the periventricular region and 2.9 (3.1; 0–14; 0; 2; 5) for the subcortical region. The group comparison showed that WMH severity was higher in the BABAD group; however, this difference was only statistically significant for the periventricular region (Table 2). *Post-hoc* Bonferroni analysis revealed that the BABAD group had a higher periventricular WMH score than the NS group.

To adjust for confounding factors, we performed multivariate analyses (Table 3). We used the 50th percentile of the WMH score as the cutoff point. To respectively compare the severity of WMH between BABAD group and NS and LAA group, we analyzed the odds ratio (OR) of greater WMH (>50th percentile of the WMH score) in NS and LAA group versus BABAD respectively. Multivariate analysis revealed that greater periventricular WMH severity was associated with age and the presence of hypertension. It also showed that the BABAD group had a higher likelihood of greater periventricular WMH severity compared to the NS and LAA groups. Subcortical WMH was only associated with aging. Although the BABAD group showed a tendency for greater subcortical WMH severity compared to the other two groups, this finding was not statistically significant.

**Table 3:** cFactors associated with severe white matter hyperintensity revealed by multivariate analyses

	Periventricular WMH ≥ 3			Subcortical WMH ≥ 2		
	OR	95% CI	*p*	OR	95% CI	*p*
Age (per year increase)	1.08	1.05–1.11	<0.001	1.04	1.02–1.07	<0.001
Sex (men versus women)	0.89	0.51–1.57	0.692	0.75	0.44–1.26	0.274
Hypertension (yes versus no)	2.66	1.46–4.82	0.001	1.30	0.75–2.26	0.352
Diabetes mellitus (yes versus no)	0.96	0.56–1.65	0.878	1.10	0.68–1.83	0.679
Hyperlipidemia (yes versus no)	0.99	0.54–1.82	0.964	0.88	0.50–1.54	0.646
Cigarette Smoking (yes versus no)	1.67	0.15–18.76	0.666	0.83	0.11–6.24	0.856
Etiology (LAA/NS versus BABAD)						
BABAD	1			1		
LAA	0.51	0.28–0.96	0.037	0.63	0.36–1.12	0.302
NS	0.40	0.21–0.74	0.004	0.74	0.42–1.31	0.302

*BABAD* basilar artery branch atheromatous disease; *LAA* large-artery >50% atherosclerotic stenosis; *NS* non-stroke subjects; *WMH* white matter hyperintensity; *OR* odds ratio; *95% CI* 95% confidence interval

Finally, we investigated whether greater WMH severity in patients with BABAD was associated with poor 3-month functional outcomes [modified Rankin Scale (mRS) > 3; Table 4]. In BABAD-associated PCS, periventricular WMH severity was associated with poor functional outcome; however, subcortical WMH did not exhibit the same relationship. Moreover, greater periventricular WMH severity (score ≥ 50th percentile) at admission could predict a poor 3-month functional outcome with an odds ratio (OR) of 3.21.

**Table 4:** Association between the severity of white matter hyperintensity and poor functional outcomes in patients with BABAD

	Model 1			Model 2		
	OR	95% CI	*p*	OR	95% CI	*p*
Periventricular WMH score ≥ 3	3.10	1.14–8.47	0.027	3.21	1.13–9.12	0.028
Subcortical WMH score ≥ 2	1.14	0.43–3.04	0.791	0.94	0.34–2.63	0.909

Model 1 = adjusted for age, sex, and stroke severity at admission (NIH Stroke Scale). Model 2 = adjusted for age, sex, stroke severity at admission, and vascular risk factors (i.e., hypertension, diabetes mellitus, hyperlipidemia, and cigarette smoking)

*WMH* white matter hyperintensity; *OR* odds ratio; *95% CI* 95% confidence interval

## Discussion

The main finding of the present study was that patients with BABAD had greater periventricular WMH severity than patients with LAA and NS. Moreover, the greater periventricular WMH severity of patients with BABAD was associated with poor 3-month functional outcomes. Following Fisher and Caplan’s pathological reports [3, 4], few studies investigated the mechanisms of BABAD. Consequently, it is currently unknown whether small vessel abnormalities other than lipohyalinosis are involved in BABAD mechanisms. Our results have provided new insights into the pathophysiology of BABAD.

In agreement with our findings, a previous study of a Chinese population revealed a very high frequency of WMH (96.4%) in patients with BABAD [15]. However, this study did not consider confounding factors, such as vascular risk factors and atherosclerotic large-artery disease, when evaluating the relationship between WMH and BABAD. WMH is associated with several vascular risk factors [7, 8, 13] and has been frequently reported in atherosclerotic large-artery disease [16, 17]. Thus, in order to validate the association between WMH and BABAD, we controlled for vascular risk factors and recruited patients with LAA for WMH comparisons. Moreover, the reduced frequency of severe atherosclerotic large-artery stenosis in patients with BABAD (Table 1) suggests that the greater periventricular WMH severity of these patients compared to patients with LAA could be attributed to other factors besides atherosclerotic large-artery disease.

Consistent with the pathological and clinical evidence for atherosclerotic large-artery disease involvement in BABAD [3, 4, 18], we also found a high frequency of severe large artery atherosclerotic stenosis (ICA and MCA) in BABAD patients. Considering WMH is a commonly used SVD marker [7–13], the vascular risk factor-adjusted finding of greater WMH severity in the BABAD group compared to the NS and LAA groups implies that SVD is also involvement in the pathophysiology of BABAD. Here we propose a “double-hit” theory concerning BABAD mechanisms. In BABAD, atheroma in the large parent artery (i.e., BA) at the opening of small penetrating vessels causes blood flow decrement (i.e., ischemia) in brainstem regions supplied by these penetrating vessels. Thus, BABAD-induced brainstem stroke may be facilitated by underlying SVD. In response to ischemia caused by large artery atheroma, penetrating vessels affected by SVD may be unable to dilate due to poor autoregulation ability. An accompany BBB damage with edema, another small vascular dysfunction, might also be involved.

Additional evidence suggests that small vessel abnormality may play a role in BABAD mechanisms. Clinical worsening (i.e., early neurological deterioration) following acute stroke, a common feature associated with lacunar infarction (i.e., small vessel occlusion infarction) [19], is also frequently reported in BABAD [18]. A recent trial found that Cilostazol, an anti-platelet drug that protects small vessel endothelium and the BBB [20], can reduce clinical worsening in BABAD [21]. This finding suggests that small vessel dysfunction may contribute to clinical worsening in BABAD. SVD involvement in BABAD pathophysiology is also supported by our finding that greater WMH severity predicts poor 3-month functional outcomes in patients with BABAD.

It is unclear why there was a difference in severity between periventricular and subcortical WMH in patients with BABAD. Different small vessel pathophysiologies have been reported for WMH in different brain regions [22]. Subcortical WMH is associated with lacunar infarction caused by small vessel lipohyalinosis [23], whereas periventricular WMH is associated with the other small vessel abnormalities such as arteriosclerosis, venous collagenosis, and perivascular edema [22, 24]. Region-specific small vessel pathophysiology again suggests that small vessel dysfunction caused by pathology other than lipohyalinotic degeneration may be involved in BABAD.

The present study has some limitations that merit discussion. Since most of our patients had brainstem infarcts, WMH rating in this region was difficult. Thus, we did not evaluate brainstem WMH, which would have been more representative of SVD in posterior circulation. Second, we did not investigate other SVD markers or small vessel function in BABAD. Future studies are needed to evaluate the role of small vessels in the pathophysiology of BABAD.

The strengths of the present study include a large patient population and validated BABAD diagnostic criteria, which were used to assess infarct location and detected plaque in the BA. Moreover, we are the first to show an association between WMH and BABAD. Greater periventricular WMH severity in patients with BABAD compared to patients with LAA and NS indicates SVD involvement in BABAD. Furthermore, greater periventricular WMH severity was an independent predictor of poor functional outcomes at 3 months after BABAD-associated PCS.

## Conclusions

We are the first to reveal a relationship between WMH and BABAD-related PCS which findings have provided insights into BABAD pathophysiology. Management strategies for BABAD should consider both large and small vessel protection.

## Abbreviations

BA: basilar artery
BABAD: basilar artery branch atheromatous disease
BAD: branch atheromatous disease
BBB: blood brain barrier
LAA: large artery atherosclerotic stenosis
mRS: modified Rankin Scale
NS: non-stroke subjects
PCS: posterior circulation stroke
SVD: small vessel disease
VBA: vertebrobasilar artery
WMH: white matter hyperintensity
